# Innate–Acquired Linkage in Immunotherapy

**DOI:** 10.3390/cells12030371

**Published:** 2023-01-19

**Authors:** Tsukasa Seya

**Affiliations:** 1 Nebuta Research Institute for Life Sciences, Aomori University, Aomori 030-0943, Japan; seya-tu@pop.med.hokudai.ac.jp; 2 Department of Vaccine Immunology, Hokkaido University Graduate School of Medicine, and Hokkaido University International Institute for Zoonosis Control, Sapporo 060-8638, Japan

The evolution of the human species is the result of genetic variation. The immune system is the mechanism that maintains the identity of the species and individuals. Innate immunity detects “non-self” nucleic acid mutations, and acquired immunity detects and eliminates “non-self” proteins. This mechanism maintains the genomic identity in individuals as well as in somatic cells. In general, non-self nucleic acids are discriminated by pattern-recognition receptors (PRRs) of innate immunity, and non-self proteins (amino acid sequences) are detected and eliminated by antibodies and T lymphocytes of acquired immunity.

Viral RNA forms double-stranded structures when replicated by virus-specific, RNA-dependent RNA polymerase in infected cells, and affects various aspects of the host cell transcription, translation, and regulation of protein expression. In addition, although nucleic acid mutations can be corrected by editing functions, RNA has a unique editing function that regulates the efficiency of protein translation.

Myeloid cells and innate lymphocytes are effectors that can be regulated during development. These cells have the unique ability to establish themselves early in embryonic development and protect host organs. Although their exact function is not yet understood, they play an important role in host survival against infection and cancer.

In this Special Issue, we focus on the innate network in the vertebrate immune system, illustrating how immune cells protect the host from various environmental factors and yet maintain host identity. It is deduced from this Special Issue that our immune system is not an isolated identity-preserving mechanism, but a functional population consisting of diverse immune cells that adapt to their environment. You can see a variety of stromal cells and mechanisms in infectious nests and tumors, which orchestrate host protection against non-self cells. 

Uehara and Takeuchi reviewed a key mechanism for RNA sensing by which host cells discriminate self from non-self nucleic acids to initiate antiviral responses reliably, including the expression of a type I interferon. Another important aspect of the RNA-mediated inflammatory response is post-transcriptional regulation. They focused mainly on this issue, particularly in regard to stem-loop RNA in inflammatory mRNAs, which act as a cis-element recognized by RNA-binding proteins (RBPs). RBPs are a family containing Regnase-1 and Roquin. The authors first described the functional concept of the RBP system. In this review, they further overviewed how post-transcriptional regulation by RBPs shapes immune reactions [1]. 

Ebihara summarized the information on helper innate lymphocytes (ILCs), which are classified into three subpopulations: T_H_1-like ILC1s, T_H_2-like ILC2s, and T_H_17/T_H_22-like ILC3s. ILCs can serve as an innate component that augments each corresponding type of acquired immunity. However, the physiological functions of ILCs are more plastic and complicated than those of expected and natural killer (NK) cells. They are affected by environmental cues and types of inflammation. The author reviewed the recent advances in understanding the interaction between ILCs and acquired immunity, including T- and B-cell responses under various conditions [2]. 

Additionally, Yasuda et al. described the relationship between ILCs and allergic diseases induced by house dust mites, particularly highlighting ILC2 in the context of the mechanism of eosinophilic airway inflammation and asthma [3]. 

Ishida described a historical background by which Dr. Honjo’s group successfully developed the programmed cell death-1 (PD-1) study. He first identified the molecule PD-1 and isolated it in the early 1990s during his long journey of investigation. Immune checkpoint inhibitors are now involved in standard therapies of cancer immunotherapy [4].

Kahn et al. summarized the possible relationship between Alzheimer’s disease and gut microbiota by explaining several factors promoting the disease. They especially focused on the therapeutic potential of natural dietary polyflavonoid anthocyanins. Gut dysbiosis and systemic inflammation appear to be critically involved in the development of neurodegenerative disorders, and the natural intake of these flavonoids may provide new therapeutic opportunities for preclinical or clinical studies [5].

In Toll-like receptor studies, Bin et al. experimentally showed that in comparison with Toll-like receptor (TLR) 4^−/−^ mice, TLR2^−/−^ mice expressed more severe pathological changes through MyD88-mediated signaling pathways during infection (mastitis) with *S. uberis*. They suggested that TLR2-mediated mitochondrial reactive oxygen species (mROS) has a significant effect on *S. uberis*-induced host defense responses in mammary glands [6]. 

Finally, our review focused on the strategy for how human dendritic cells are targeted to evoke acquired immunity. The review mentioned that a TLR3 adjuvant, Adjuvantic RNA for DC-targeting (ARNAX), nicely fits for this purpose. ARNAX works as a noninflammatory adjuvant that harmlessly evokes the activation of antigen-presenting dendritic cells, which is followed by antigen-specific T-cell proliferation and antibody production. This review discusses the challenges faced in the clinical development of novel adjuvants [7].
